# Supplementary figures and images for: Predictive value of neutrophil to lymphocyte ratio for clinical outcomes in liver cirrhosis: A systematic review and meta-analysis
Source: PLoS One. 2025 Nov 4;20(11):e0335925. doi: 10.1371/journal.pone.0335925 (PMC12585051; doi:10.1371/journal.pone.0335925)

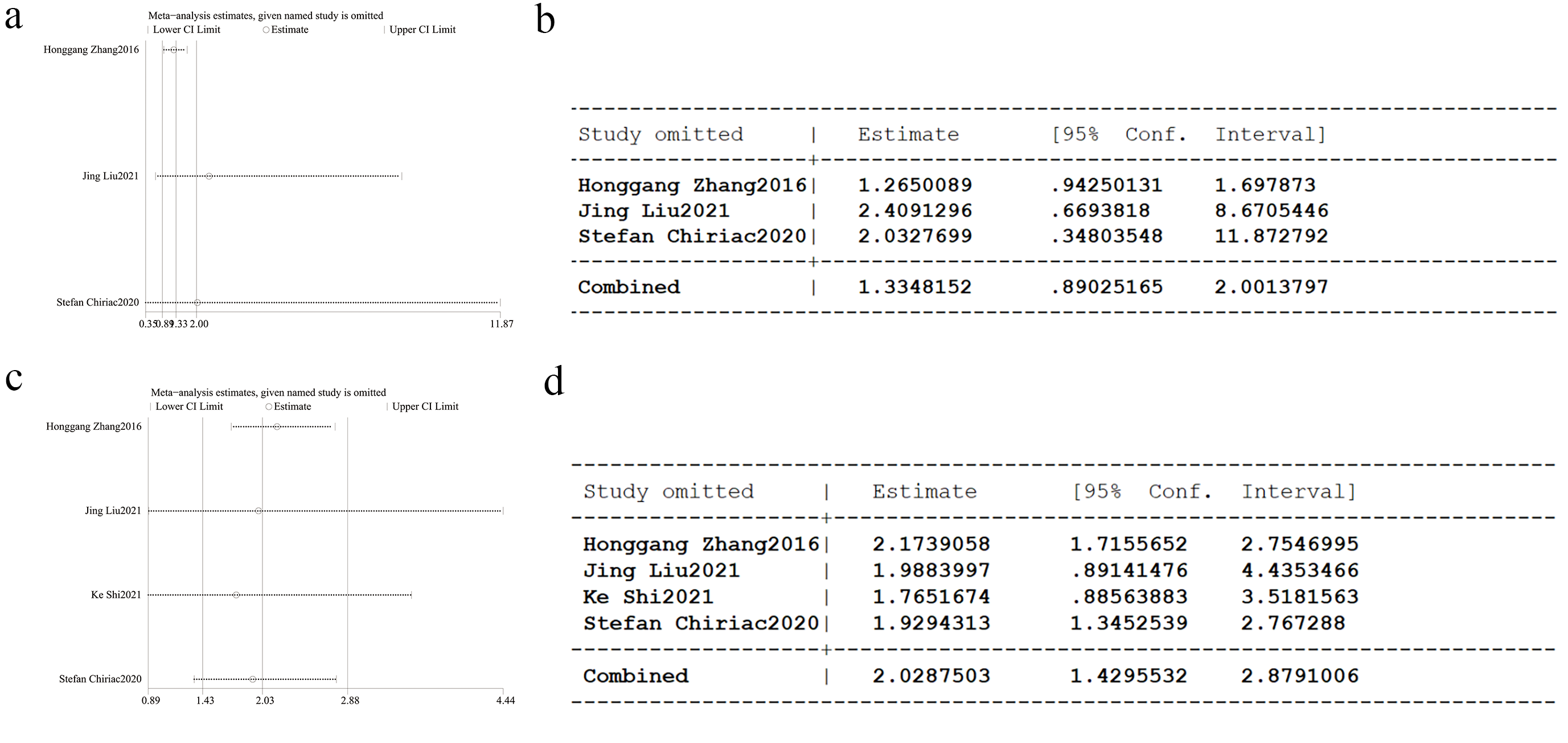

Supplement: S1 Fig — (TIF) [file pone.0335925.s001.tif]

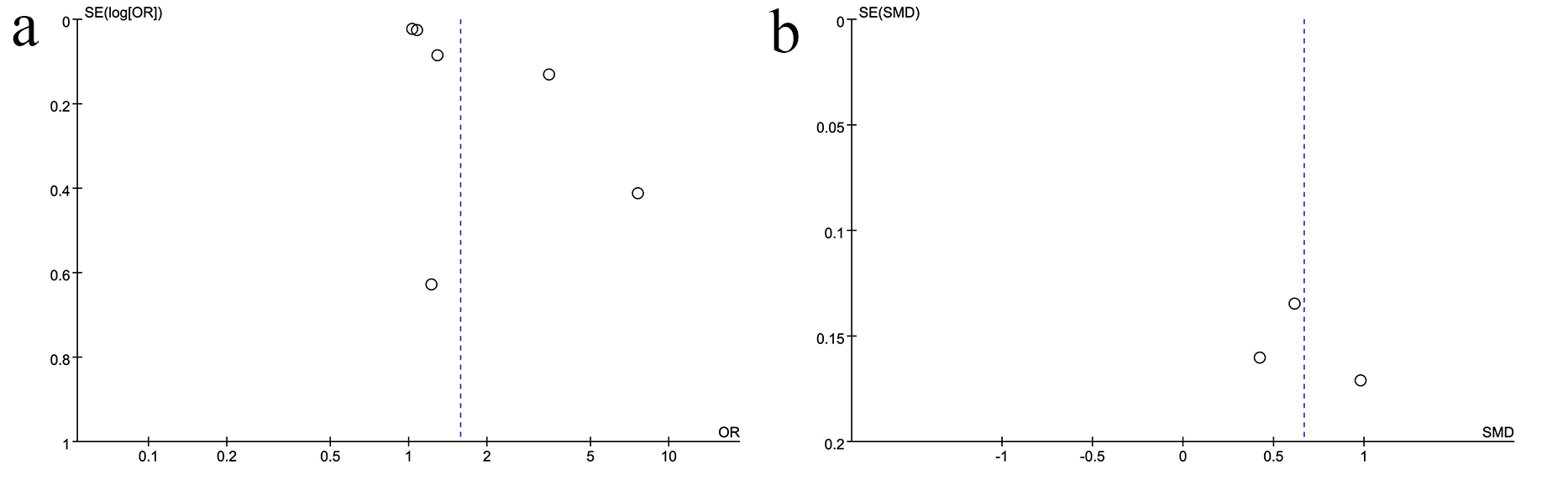

Supplement: S2 Fig — (TIF) [file pone.0335925.s002.tif]

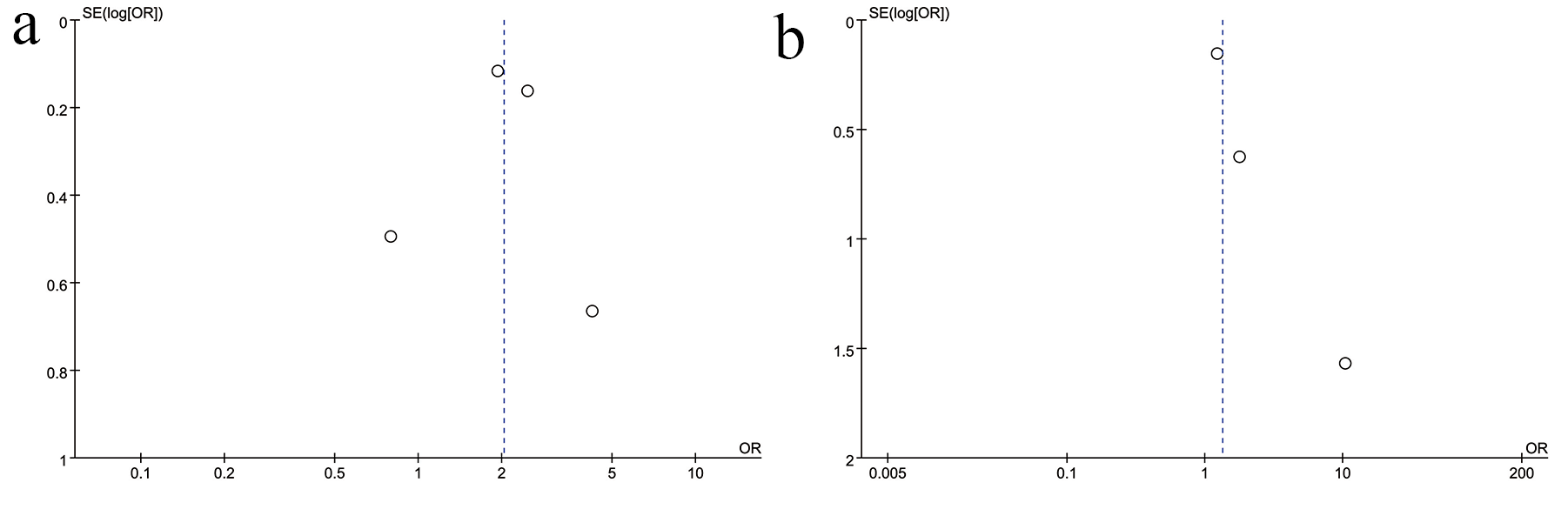

Supplement: S3 Fig — (TIF) [file pone.0335925.s003.tif]
