# Supplementary material for: Predictive value of neutrophil to lymphocyte ratio for clinical outcomes in liver cirrhosis: A systematic review and meta-analysis
Source: PLoS One. 2025 Nov 4;20(11):e0335925. doi: 10.1371/journal.pone.0335925 (PMC12585051; doi:10.1371/journal.pone.0335925)
Supplement: Attachment — Submitted filename: Systematic Review Paper.docx [file pone.0335925.s006.docx]

**Peer Review Report**

Peer Review of a journal article: Predictive value of neutrophil to lymphocyte ratio for the clinical outcomes of liver cirrhosis: a systematic review and meta-analysis.

**Idowu Peter Shileayo Adebayo.**

**Overview**

Thank you for an opportunity to peer-review this article titled Predictive value of neutrophil to lymphocyte ratio (NLR) for the clinical outcomes of liver cirrhosis: a systematic review and meta-analysis. This systematic review addresses the importance of NLR as a useful biomarker to determine the prognosis of liver cirrhosis. The authors have made a significant effort in collecting and synthesizing the evidence. However, few areas require clarification or improvement to enhance transparency and methodological rigor.

**Introduction**

This section is well captured and provides adequate background and literature review for the study. It also identifies and addresses the knowledge gap in the existing literature on the prognostic predictive effects of NLR on liver cirrhosis.

**Methodology**

The sample size is adequate, and the sampling method is well-explained. The authors should please include the full search strings of the databases used in the appendix for reproducibility. Also, they should try and provide or show the table that has the complete search syntax as contained in the last sentence under **‘2.1** ***Literature Search Methodology’.***

Under Inclusion and Exclusion criteria, the authors excluded Neonatal or Pediatric studies. However, Pediatric sepsis studies made up two (2) out of eighteen (18) of the final analyzed studies for the paper. Please, let the authors clarify these two areas (line 6 under **2.2 Inclusion and exclusion criteria** and line 5 under **3.1 Identification of relevant studies**).

**Results**

The review adequately addressed the risk of bias and quality assessment with the appropriate tools being used (Newcastle-Ottawa Scale). This is good! A meta-analysis was used, but heterogeneity is high (I² =89%). The authors should consider providing a more in-depth exploration of sources of heterogeneity or revisiting whether the meta-analysis is appropriate.

**Discussion**

Adequate discussion of the result analysis.

**Limitation**

The authors’ effort to acknowledge the study’s limitations is appreciated, as this is a crucial aspect of transparent research reporting. The limitations section acknowledges moderate heterogeneity of the study, potential publication bias and the effects of pharmacotherapy on the NLR. I would recommend if the authors could add the possibilities of tackling these limitations and how these can guide future research on the prognostic relevance of NLR for liver cirrhosis.

**Conclusion**

This manuscript is well-written and adequately structured. The contents are relevant and adequate for publication.

**Minor Comments:**

-The authors should please clarify acronyms (PRISMA) at first mention (line 8 under subsection *2.2 Inclusion and exclusion criteria*).

-The authors should cite appropriately with adequate footnotes to all the pictures at the appendix.

**Recommendations**

-This is a good paper for publication.

-The authors should look into the comments and make adjustments where necessary.

I recommend that this article be accepted for publication after the relevant minor corrections must have been made.
